# A single-center experience with the laparoscopic Warshaw technique in 122 consecutive patients

**DOI:** 10.1007/s00464-015-4720-x

**Published:** 2016-01-07

**Authors:** Hanbaro Kim, Ki Byung Song, Dae Wook Hwang, Jae Hoon Lee, Sang Hyun Shin, Eun Sung Jun, Seong-Ryong Kim, Bong Jun Kwak, Tae Gu Kim, Kwang-Min Park, Young-Joo Lee, Song Cheol Kim

**Affiliations:** Division of Hepatobiliary and Pancreatic Surgery, Department of Surgery, University of Ulsan College of Medicine and Asan Medical Center, 88 Olympic-ro 43 gil, Songpa-gu, Seoul, 05505 Korea

**Keywords:** Warshaw operation, Distal pancreatectomy

## Abstract

**Background:**

Preservation of the spleen in distal pancreatectomy has recently attracted considerable attention. Our current study aimed in the first instance to define the safety of lap-WT in relation to the capacity of this technique to achieve preservation of the spleen and secondly to investigate the effectiveness of a planned lap-WT procedure or early conversion to lap-WT in selected patients with a large tumor attached to the splenic vessels.

**Methods:**

Among 1056 patients who underwent a laparoscopic distal pancreatectomy between January 2005 and December 2014 at our hospital, 122 (24.6 %) underwent lap-WT which were analyzed. The 122 patients were categorized into two groups chronologically (early group: 2005–2012, late group: 2013–2014).

**Results:**

The median follow-up was 35 months, and the median operation time was 181 min. The median postoperative hospital stay was 7 days, and the median estimated blood loss was 316 ml. Postoperative complications occurred in 9 patients (7.3 %), including 4 patients (3.2 %) with major pancreatic fistula (ISGPF grade B, C). A reoperation to address postoperative bleeding was needed in one patient. During a median follow-up of 35 months, there were no clinical significant splenic infarctions or gastric varices in any case. All patients were observed conservatively. In patients in the late group who underwent the lap-WT, the mean operating time (171 vs. 205 min, *p* = 0.001) and mean estimated blood loss (232.1 vs. 370.0 ml, *p* = 0.017) were significantly less than the early group cases who received lap-WT.

**Conclusions:**

A lap-WT is a safe treatment strategy in select cases when used as a way of preserving the spleen. When splenic vessel preservation is technically challenging, for example when the tumor is enlarged or is attached to the splenic vessels, planned lap-WT or early conversion to lap-WT may be a feasible option.

Laparoscopic distal pancreatectomy (LDP) is a standard treatment for benign or low-grade malignant pancreatic tumors of the left-sided pancreas. To reduce the risk of post-splenectomy sepsis and late hematologic disorders and malignancy [1, 2], splenic preservation during distal pancreatectomy (SPDP) for benign or low-grade malignant disease of pancreas has been proposed. There are two spleen-preserving SPDP methods: In one, the splenic artery and vein are preserved, and in the other they are excised. However, preservation of the splenic artery and vein while dividing the small branches to the distal pancreas is technically more challenging. In contrast, a spleen-preserving distal pancreatectomy involving ligation of both splenic vessels (the Warshaw procedure) [3, 4] can be performed safely and effectively.

LDP for the treatment of benign or low-grade malignant pancreatic body and tail lesions commenced in our hospital in 2005 [5]. In our previous study, we demonstrated that the laparoscopic Warshaw technique (lap-WT) can be used for patients with large and benign or low-grade malignant tumors that distort and compress the vessel course and that this approach yields acceptable clinical outcomes compared to splenic vessel-preserving LDP (SVP-LDP) [6]. In our current study, we aimed to use these previous findings as the basis for an assessment of the long-term, clinically significant complications associated with lap-WT and thereby evaluate the safety of this method of preserving the spleen.

We performed SVP-LDP at our hospital from 2005 to 2012 to treat benign masses in the pancreatic body or tail. If this surgery was not successful due to the presence of either a relatively large tumor or splenic vessel involvement, we converted to lap-WT. However, this strategy was time consuming and was also associated with considerable blood loss. We therefore adopted a new approach from 2013 to 2014 in which we introduced a much earlier conversion to lap-WT after a failure of SVP-LDP and performed planned lap-WT procedures in selected patients because of the presence of either a large tumor or a tumor attached to the splenic vessels. The second purpose of our current study was to compare the clinical outcomes between patients who underwent lap-WT after SVP-LDP failure and patients who received a planned lap-WT or early conversion to lap-WT.

## Materials and methods

### Patients

From November 2005 to December 2014, a total of 1056 patients underwent laparoscopic distal pancreatectomy (LDP) at our institution (Asan Medical Center, Seoul, Korea). Of these cases, 495 patients with benign or low-grade malignant lesions in the body and tail of the pancreas underwent spleen-preserving LDP among which 122 patients underwent lap-WT for spleen preservation. We further examined these 122 cases in our present study. A spleen preservation approach had not been considered appropriate among the initial 1056 LDP patients with (1) malignancies on preoperative imaging, (2) severe pancreatitis with or without pseudocyst, or (3) a relatively large tumor located at the hilum of the spleen. Where a malignancy was suspected, a splenectomy was always performed. The 122 patients selected for analysis in this study were categorized into two groups, according to their treatment chronology. The early group (EG) included patients who underwent a conversion to lap-WT between 2005 and 2012 following an SVP-LDP failure. The late group (LG) comprised patients who underwent either planned or early conversion to lap-WT from 2013 to 2014. Demographic data (age, gender, body mass index, and tumor size), operating procedures and times, postoperative complications, length of hospital stay, and pathologic diagnoses were retrospectively compared between the two groups. The actual spleen preservation rate, which is the ratio of practical spleen and intended spleen preservation, was also compared between the two groups.

### Surgical procedure and strategy

**Fig. 1:**
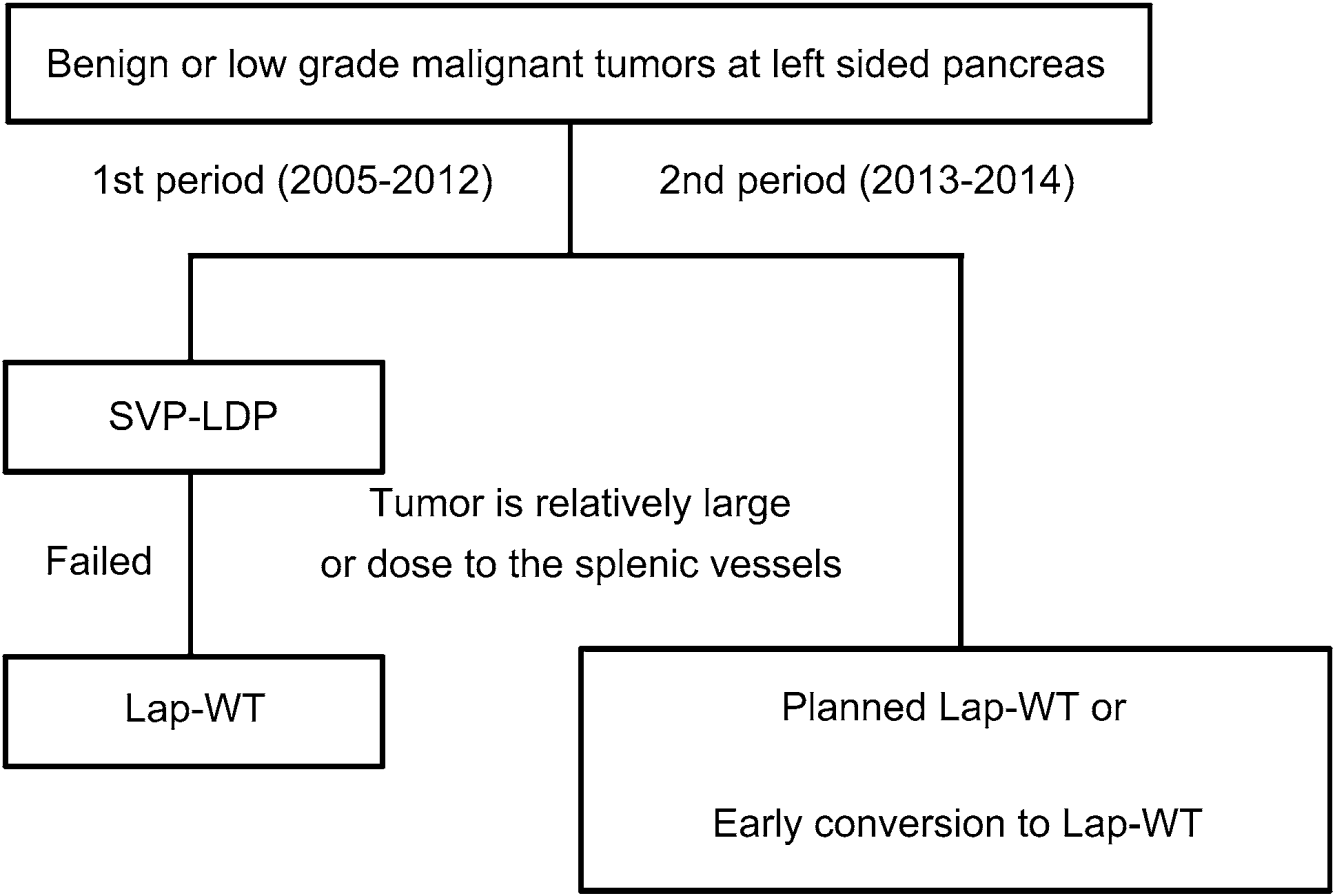
Laparoscopic surgical treatment strategies for the patients with benign or low-grade malignant tumor at *left*-*sided* pancreas. *SVP*-*LDP* splenic vessel-preserving laparoscopic distal pancreatectomy, *lap*-*WT* laparoscopic Warshaw technique

In all of our current study patients, an open technique was used to establish pneumoperitoneum through a periumbilical 12-mm trocar used under direct vision. Abdominal pressure was maintained at 12 mmHg by insufflation of CO_2_ gas. This surgical technique has been previously described in a report from our institution [5]. During the EG period, SVP-LDP was the first treatment choice for a benign pancreatic mass at the pancreatic body and tail. The decision to perform SVR was made intraoperatively, as only then was it possible to determine whether dissection of the pancreas from the splenic vessels could be achieved or whether uncontrolled bleeding was occurring during SVP-LDP (Fig. 1). The lesser sac was accessed via the gastrocolic omentum outside of the gastroepiploic arcade, which was carefully preserved by the surgeon. Retraction of the stomach and visualization of the body and tail of the pancreas and the splenic hilum were enabled by division of the omentum. Dissection was stopped before the short gastric vessels were reached to protect them from possible injury. The avascular plane behind the body and tail was reached by opening the retroperitoneum along the inferior margin of the pancreas. The splenic artery was then controlled and divided.

Prior to splenic vessel ligation, we used a vascular clamp to confirm the color of the spleen. When splenic vessels are clamped, the spleen color is sometimes darker than it had been before the vascular interruption. Nonetheless, a dark red color indicative of perfusion and viability should still be evident. An area of likely necrosis is indicated by a sharply demarcated area, dark gray or black in appearance. This finding may indicate the need for removal of the spleen, depending on the volume of critical ischemia. Partial areas of apparent infarction, perhaps less than 30–40 % of the spleen, do not preclude the use of SVR. However, during the LG period, if the tumor was found to be either relatively large or close to the splenic vessels on preoperative CT imaging, then planned or early conversion to lap-WT was performed (Fig. 1). Also, if splenic infarction of more than 50–70 % of the spleen was identified intraoperatively, then lap-WT was performed without splenectomy, but only if it was certain that the short gastric artery and the gastroepiploic artery could be preserved.

After the splenic artery was clipped and divided, the inferior border of the pancreas was then freed from the transverse mesocolon all the way to the tip of the pancreatic tail. Once the distal retroperitoneal dissection reached the splenic hilum, the splenic vascular pedicle was ligated and divided between the tip of the pancreas and the splenic hilum. Next, the tail of the pancreas was moved to the right, the splenic vein was divided, and the pancreas was transected and removed. The color and viability of the spleen was reconfirmed before closure of the abdomen.

### Complications and follow-up

Preoperative and postoperative computed tomography (CT) images were compared to evaluate postoperative abdominal changes, splenic perfusion, and whether vessels were native or collaterals that developed after surgery. Follow-ups included a thorough medical examination and a contrast-enhanced CT scan at 3 months and then once yearly assessments following the index operation to assess the presence of gastric varices and splenic perfusion, and any additional postoperative complications. Complications were classified according to the criteria of Clavien and Dindo [7]. In accordance with the 2005 International Study Group of Pancreatic Fistula (ISGPF) [8], each pancreatic fistula was classified as grade A, B, or C [9].

Each radiologic study was reviewed to assess for splenic infarcts and gastric varices. Splenic infarction volumes were measured on postoperative CT scan at 3 days, 3 and 12 months by a single, expert radiologist. Splenic perfusion was grouped into grades on the basis of the percentage of the total splenic volume affected by the infarction: grade 0, intact; grade 1, <30 % infarction; grade 2, 30–50 % infarction; grade 3, 50–70 % infarction; and grade 4, >70 % infarction. When tortuous vascular structures larger than 5 mm in diameter were seen, gastric varices were diagnosed on CT images by the expert radiologist who assessed the splenic infarction volume. Gastric varices located along the outside border of the gastric wall were defined as perigastric varices, and those existing within the wall of stomach were designated as submucosal gastric varices. Gastric varices were classified into perigastric and submucosal gastric type. Perigastric varices were graded based on measurement of diameter: grade 1, 5–10 mm; grade 2, 10–20 mm; and grade 3, >20 mm.

### Statistical analysis

Continuous variables were expressed as median and range or mean ± standard deviation. Categorical variables were reported as numbers and percentages. The AX2 test (with Yates continuity correction in a 2 × 2 contingency table) was used for nominal data. Numerical variables were compared using a Student’s *t* test. Data were considered significant at *p* < 0.05. SPSS statistical software ver. 17.0 (SPSS, Inc., Chicago, IL) was used for all statistical analyses.

## Results

### Operative methods and actual spleen preservation rate

Essential characteristics of the surgical procedures used in the current patient series are summarized in Table 1. Of the 1056 patients who underwent laparoscopic distal pancreatectomy at our hospital, 561 patients (52.1 %) underwent a laparoscopic distal pancreatectomy with splenectomy (LDPS), 373 patients (35.3 %) underwent a SVP-LDP, and 122 patients (11.6 %) underwent a lap-WT. The actual spleen preservation rate was 496/613 patients (80.9 %).

**Table 1:** Surgical procedures and actual spleen-preserving rate

	Patients (*n* = 1056) %
*Type of surgery*	
LDPS	561 (52.1)
SVP-LDP	373 (35.3)
Lap-WT	122 (11.6)
Actual spleen-preserving rate*	496/613 (80.9)

*LDPS* laparoscopic distal pancreaticosplenectomy, *SVP*-*LDP* splenic vessel-preserving laparoscopic distal pancreatectomy, *lap*-*WT* laparoscopic Warshaw technique

* The actual spleen preservation rate, which is the ratio of practical spleen and intended spleen preservation

### Patient characteristics

Table 2 lists the patient characteristics and the pathologic diagnoses for the 122 cases that underwent lap-WT. The median age of this lap-WT cohort was 48 years (range 38–58), and 83.6 % of the patients were female. The mean tumor size was 4.1 ± 2.3 cm, and BMI was 23.1 ± 2.9 kg/m^2^. The most common diagnosis was a solitary pseudopapillary neoplasm (38 patients, 31.2 %).

**Table 2:** Characteristics of patients who underwent lap-WT

	Patients (*n* = 122)	
	Number	%
*Age at diagnosis (year)*		
Median (range)	48 (range 38–58)	
*Sex*		
Men	20	16.4
Women	102	83.6
BMI (kg/m^2^, mean ± SD)	23.1 ± 2.9	
Tumor size (cm, Mean ± SD)	4.1 ± 2.3	
*Pathologic diagnosis*		
Endocrine tumors	10	8.1
MCN	30	24.6
SCN	15	12.4
IPMN	18	14.7
SPN	38	31.2
Cyst	1	0.8
Pseudocyst	5	4.2
Malignancies	3	2.4
Other diagnosis	2	1.6

*MCN* mucinous cystic neoplasm, *SCN* serous cystic neoplasm, *IPMN* intrapapillary mucinous neoplasm, *SPN* solitary pseudopapillary neoplasm, *SD* standard deviation

### Perioperative outcomes

Perioperative outcomes are outlined in Table 3. There were no perioperative mortality and no conversion to open surgery among the lap-WT patients. The median operation time for these 122 patients was 181.5 min (range 144–220), and the median estimated blood loss was 316.7 ml (range 120–493). The median postoperative hospital stay was 7 days (range 6–9). Based on the Clavien–Dindo classification, postoperative complications occurred in 9 (7.3 %) patients, including 3 (2.5 %) cases with intra-abdominal fluid collection and 4 (3.3 %) patients with grade B or C pancreatic fistula. On postoperative day 1, one of the study patients underwent an early reoperation by splenectomy to control for a postoperative hemorrhage. Another patient underwent a reoperation on postoperative day 10 to treat a small bowel strangulation from an incisional hernia. According to the ISGPF definition, 55 (45.1 %) of our lap-WT patients had pancreatic fistulas. These were classified as grade A (*n* = 51; 41.8 %), B (*n* = 3; 2.5 %), or C (*n* = 1; 0.8 %). A single patient (grade C) showed postoperative fluid collection and underwent endoscopic ultrasonography (EUS)-guided gastrocystic drainage.

**Table 3:** Perioperative outcomes

	Patients (*n* = 122)
Median operative time (min, IQR)	181.5 (range 144–220)
Median estimated blood loss (ml, IQR)	316.7 (range 120–493)
Median postoperative hospital stay (day, IQR)	7.0 (range 6–9)
Clavien–Dindo classification	
I	1 (0.8 %)
II	5 (4.1 %)
IIIa	1 (0.8 %)
IIIb	2 (1.6 %)
Pancreatic fistula (ISPGF)	
Grade A	51 (41.8 %)
Grade B	3 (2.5 %)
Grade C	1 (0.8 %)
Conversion rate (%)	0
Postoperative bleeding	1 (0.8 %)

*IQR* interquartile range, *ISPGF* International Study Group on Pancreatic Fistula

### Long-term clinical outcomes

**Fig. 2:**
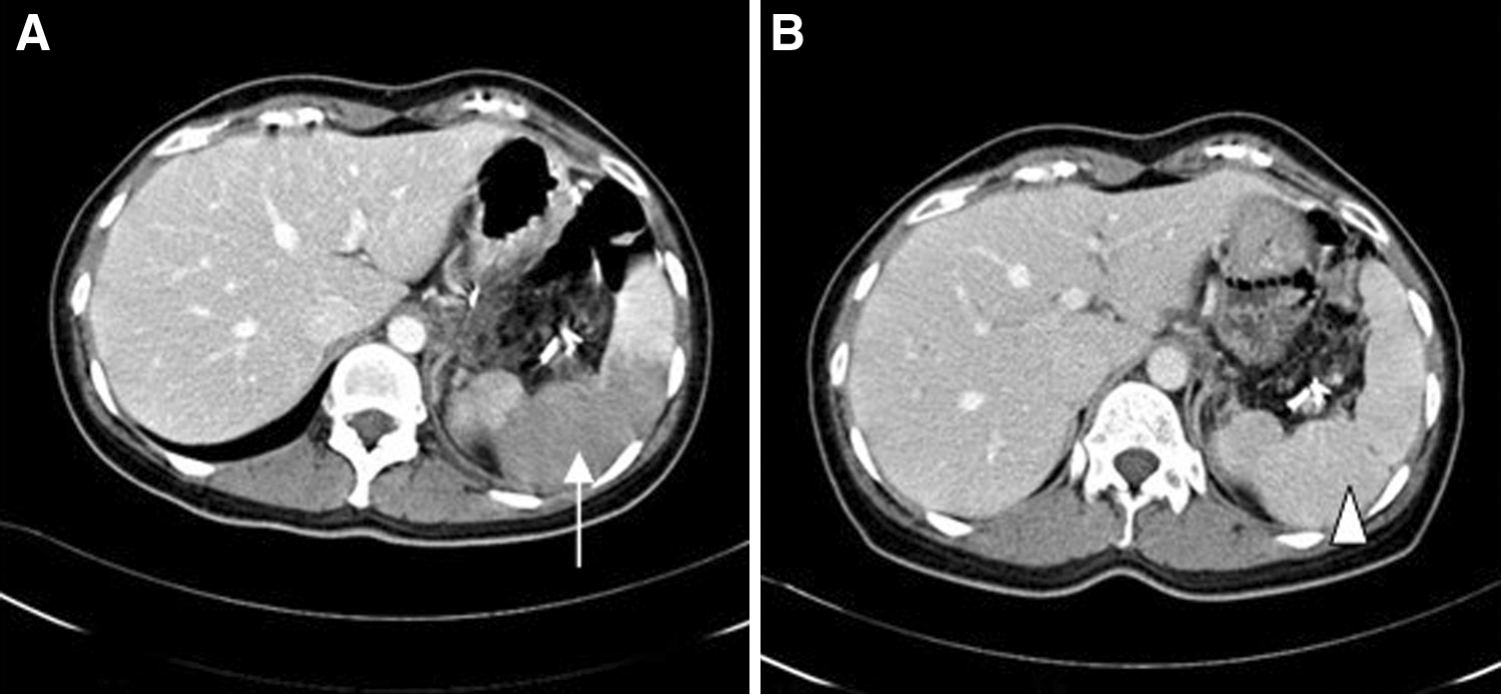
A patient with a grade 3 splenic infarction following lap-WT. A lap-WT was performed in this case to treat SPN at the body of the pancreas. **A** The CT scan showed a splenic infarction of greater than 50 % on postoperative day 3 (*white arrow*). **B** This recovered fully within 3 months, as indicated on follow-up CT scan (*white arrow head*)

**Fig. 3:**
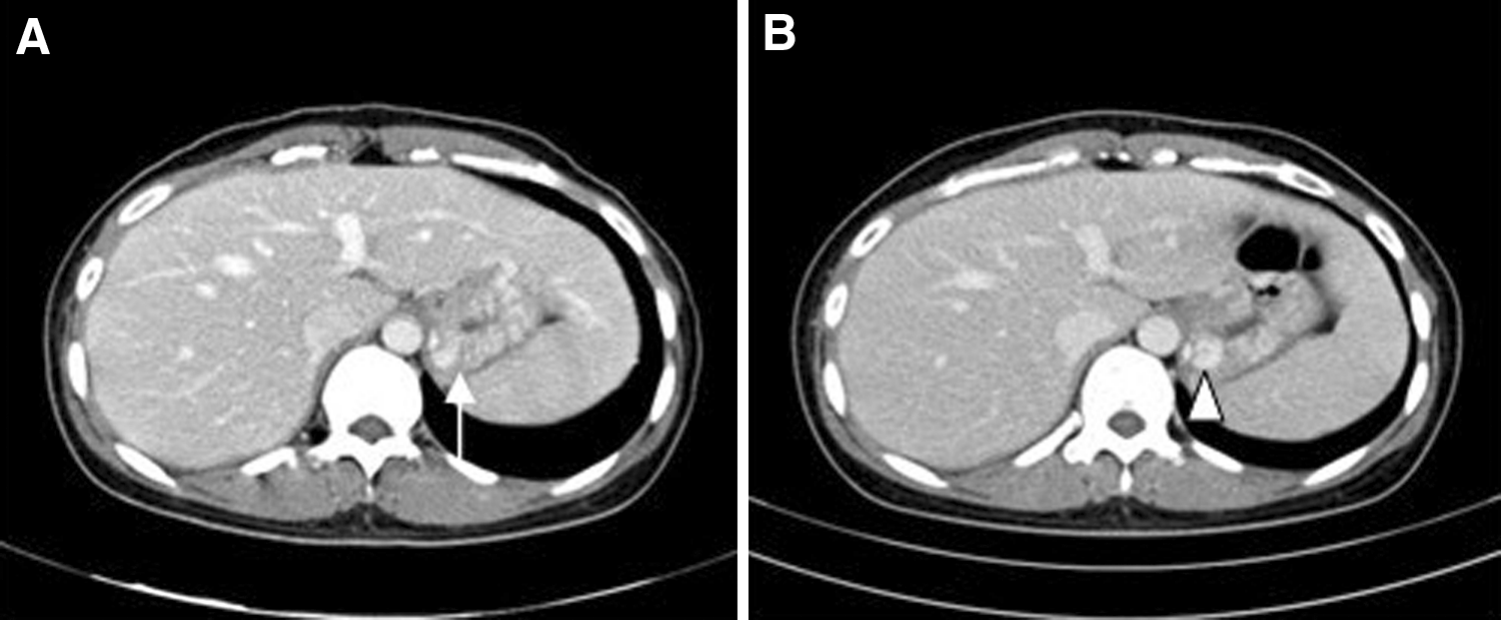
**A** A patient with grade 2 perigastric varices following lap-WT revealed at the 12-month follow-up by CT scan (*white arrow*). **B** At 4 years postoperatively, perigastric varices above 10 mm were still evident in this patient, but there were no symptoms and no gastrointestinal bleeding (*white arrow head*)

Table 4 lists the splenic infarction and gastric varices outcomes in our study cohort. On postoperative day 3, the rate of splenic infarction by CT scan was 54.2 % (66/122), and this included grade 1 (*n* = 30; 24.6 %), 2 (*n* = 18; 14.8 %), and 3 (*n* = 18; 14.8 %). No specific treatment for this complication was administered. On follow-up CT scan taken within 3 months of the operation, 54/66 (81.8 %) patients with a splenic infarction recovered, while 62 of these cases (94.0 %) had recovered within 12 months (Fig. 2). The remaining 4 patients recovered partially to an infarction volume of below 10 % of the spleen. During a median follow-up of 35 months (IQR 19–60), there were no clinically significant splenic infarctions in our lap-WT cohort. All patients were observed conservatively. At 12 months post-surgery, a follow-up CT scan revealed perigastric varices in 25 patients (20.5 %) ranging from grade 1 (*n* = 22; 18.0 %) to grade 2 (*n* = 3; 2.5 %). No grade 3 varices were identified (Fig. 3). Submucosal gastric varices also were detected in 10 patients (8.1 %) on CT scans performed 12 months after surgery. During the follow-up period, there were no instances of gastrointestinal bleeding from these perigastric varices and submucosal gastric varices.

**Table 4:** Long-term clinical outcomes

	Patients (*n* = 122)	
	Number	%
Follow-up duration (month, median, IQR)	35 (19–60)	
Splenic infarction on 3 days	66	54.2
Grade 1 (0–30 %)	30	24.6
Grade 2 (30–50 %)	18	14.8
Grade 3 (50–70 %)	18	14.8
Grade 4 (70–100 %)	0	0
Recovery on 3 months	54/66	81.8
Recovery on 12 months	62/66	94
Perigastric varices	25	20.5
Grade 1 (5–10 mm)	22	18
Grade 2 (11–20 mm)	3	2.5
Grade 3 (21 mm-)	0	0
Submucosal gastric varices	10	8.1
Gastrointestinal bleeding	2	0
Late complication	10	8.1

*IQR* interquartile range

### Clinical outcomes between the early group (EG) and the late group (LG) of lap-WT patients

Table 5 compares the outcomes in the EG and LG groups of lap-WT cases. The median operative time (171.0 vs. 205.0 min, *p* = 0.001) and median estimated blood loss (232.1 vs. 370.0, *p* = 0.017), respectively, were significantly different between these two groups. The overall rate of complications did not differ between the EG and LG patients (*p* = 0.377), and no statistically significant difference was found between the occurrence of a pancreatic fistula (*p* = 0.76). In addition, no difference was evident between the groups in the rates of splenic infarction (*p* = 0.742) or perigastric varices (*p* = 0.448).

**Table 5:** Comparision of the outcomes between the early and late lap-WT patient groups

	EG (2005–2012) (*n* = 61)	LG (2013–2014) (*n* = 61)	*p* value
Median operative time (min, IQR)	205.0 (162–239)	171.0 (131–198)	0.001
Median estimated blood loss (ml, IQR)	370.0 (210–599)	232.1 (45–426)	0.017
Median postoperative hospital stay (days, IQR)	7.0 (6–8)	7.0 (6–9)	0.169
Postoperative complication	4 (6.6 %)	5 (8.1 %)	0.377
Pancreatic fistula (ISPGF grade B/C)	2 (3.3 %)	2 (3.3 %)	0.76
Late complication	6 (9.8 %)	4 (6.6 %)	0.393
Splenic infarction	35 (57.3 %)	31 (50.8 %)	0.742
Gastric varices	13 (12.3 %)	12 (19.4 %)	0.448

*IQR* interquartile range, *ISPGF* International Study Group on Pancreatic Fistula

## Discussion

A splenectomy is associated with a lifetime risk of developing overwhelming post-splenectomy infection (OPSI), hematologic disorders, and malignancies [10, 11]. Hence, spleen preservation during surgery is highly desirable. A spleen-preserving distal pancreatectomy is thus becoming the preferred approach for the treatment of pancreatic diseases involving benign or low-grade tumors. This approach involves conservation of the splenic artery and vein and is comparatively easy and safe in patients without severe pancreatitis or a pseudocyst and who do not have a relatively large tumor located at the hilum of the spleen [12]. This operation preserves the splenic artery and vein by dissecting and carefully ligating the multiple, small, short branches of the splenic vessels that extend to the body and tail of the pancreas. Notably, however, dissecting the splenic vessels from the pancreas may be difficult if a large tumor has attached to the course of these vessels. This may be especially challenging when a laparoscopic technique is used. In patients for whom preservation of the splenic vessels will be technically difficult, it may be prudent to use lap-WT. In this procedure, the splenic artery and vein are resected so that the short gastric and left gastroepiploic vessels are retained to preserve the blood supply to the spleen.

Among the concerns in relation to adverse outcomes following lap-WT are spleen-related complications, particularly splenic infarction. Ferrone et al. [3] have reported that only 3 of 156 patients (1.9 %) who underwent WT required a reoperation for a splenic infarction from 3 to 100 days postoperatively, and that abdominal pain and/or fever had led to these surgeries. Kohan et al. [13] reported that some degree of splenic hypoperfusion could be observed at 1 week post-operation in 63 % (22/35) of their patients who underwent lap-WT. Matsushima and colleagues reported that although lap-WT induced a splenic infarction in 24 % (4/17) of their patients, all of those infarctions were focal and none required a splenectomy. In all of the patients in that study, the infarcted volume was less than 50 % of the spleen [14]. In our present study, the splenic infarction rate after lap-WT was 54.2 % (66/122). Infarction of more than 50 % of the spleen (grade 3) was found in 18 (14.8 %) of our lap-WT patients on CT imaging on postoperative day 3. Our review of CT images further showed that splenic perfusion had returned to normal within 3 months postoperatively in 54/66 (81.8 %) patients and within 12 months in 62/66 (94.0 %) patients. Warshaw reported that splenic infarction does not require an intervention as long as less than one-third of the spleen is affected [15]. Our present results suggest that splenic infarction affecting more than 50 % of the spleen after lap-WT is acceptable because most of these patients will recover within 3 months. However, as it is essential that the gastroepiploic arcade be carefully preserved during lap-WT surgery, the dissection is stopped before the short gastric vessels were reached to avoid any injury to the short gastric artery. However, if such preservation of the short gastric and gastroepiploic vessels creates any uncertainty, it may not be safe to attempt to preserve the spleen by lap-WT.

In clinical practice, gastric varices frequently occur in patients with SVR. Following ligation of the splenic artery and vein, the spleen is supplied by the increased blood flow through the short gastric and left gastroepiploic vessels. This increased blood flow leads to the gastric varices along the gastric wall and, as a consequence, can result in gastrointestinal bleeding. From a clinical point of view, it is important to recognize that submucosal gastric varices may hemorrhage. Perigastric varices also may form into submucosal gastric varices after penetrating the muscularis externa [16, 17]. In our current study series, perigastric varices were found in 22 patients (18.0 %), and in 3 cases (2.5 %) these were greater than 10 mm in diameter. Submucosal gastric varices also were found in 10 patients (8.1 %). However, none of our current study patients with perigastric varices and submucosal gastric varices developed gastrointestinal bleeding during the follow-up period. Notably, a previous study from our center [15] also reported no instances of gastrointestinal bleeding from gastric varices during a follow-up period of 31 ± 15 months. Ferrone et al. [3] reported following 125 patients for up to 21 years with no bleeding from perigastric varices. Miura et al. [18, 19] did report a single patient with gastrointestinal bleeding after lap-WT, but still concluded in 2011 that lap-WT is feasible. However, we think that patients with not only submucosal gastric varices but also perigastric varices need further long-term follow-up with serial CT scan.

In our present study, the median operative blood loss and median operative time during lap-WT were 316.7 ml (range 120–493) and 181.5 min (range 144–220), respectively. However, the median operative time for the LG patients was significantly shorter than that of the EG cases (171.0 vs. 205.0 min, *p* = 0.001). The median estimated blood loss in the LG group also was significantly less than that in the EG patients (232.1 vs. 370.0 ml, *p* = 0.017).

Initially, we planned to perform SVP-LDP for the patients in the EG group and thus did not intend to perform lap-WT to treat the lesions of the body and tail of the pancreas in these cases. During the EG period (2005–2012), when SVP-LDP was our main treatment focus, we converted to lap-WT only when intraoperative bleeding occurred or when we found it difficult to preserve splenic vessels, due to the presence of a large tumor or of a tumor attached to a splenic vessel. However, the strategy we used during this period was found to be time consuming and could also entail a great deal of blood loss. Starting in 2013, we adopted a strategy that incorporated either pre-planned lap-WT or an early conversion to this method. This strategy was adopted because we had found it possible to achieve acceptable clinical outcomes using lap-WT both for patients with large and benign tumors and in cases of low-grade malignant tumors that distort and compress the course of the vessels [6]. After we had altered our surgical strategy, we achieved improved outcomes in terms of both the median blood loss and median operative time. Our actual rate of spleen preservation with this new protocol has been 80.9 %, and this rate has been increasing since 2013. Nevertheless, SVP-LDP appears to be the preferable technique. The blood supply to the spleen is well maintained, and the danger of splenic necrosis and abscess formation is reduced by preserving splenic artery and vein. Therefore, this procedure should be performed when possible, and for cases of presence of a large tumor or tumor being attached to a splenic vessel, it should be switched to a lap-WT [6, 20].

In conclusion, lap-WT is a safe and feasible procedure to treat lesions of the pancreas and preserve the important immunological functions of the spleen. In particular, a pre-planned lap-WT or early conversion to lap-WT may prove to be available option for patients for whom the preservation of splenic vessels is technically challenging.
